# Predictors of Poor Mental Health Outcomes in Healthcare Workers during COVID-19: A Two Waves Study

**DOI:** 10.3390/healthcare12191921

**Published:** 2024-09-25

**Authors:** Emanuela Saveria Gritti, Giulia Bassi, Arianna Schiano Lomoriello, Alessandra Simonelli, Silvia Salcuni, Tommaso Boldrini, Paolo Girardi

**Affiliations:** 1 Department of Psychology, University of Milano-Bicocca, 20126 Milano, Italy; 2 Department of Developmental Psychology and Socialization, University of Padova, Via Venezia 8, 35131 Padova, Italy; 3 Department of Cognitive System, Denmark Technical University (DTU), 2800 Copenhagen, Denmark; 4 Department of Environmental Sciences, Informatics and Statistics, University Ca’ Foscari of Venice, 30172 Venice, Italy

**Keywords:** COVID-19, cross-sectional study, lifetime traumatic events, healthcare workers, poor mental health

## Abstract

**Objective:** This cross-sectional study aimed to identify potential predictors of poor mental health outcomes among healthcare workers in two different waves of the COVID-19 emergency in Italy. **Methods:** An online survey collected data from N = 557 healthcare workers (21–77 years). The study predictors were sociodemographic characteristics, occupational status, factors related to the work environment, COVID-19-related adverse events, and lifetime traumatic events. The poor mental health outcomes that were considered were depersonalization/derealization, anxiety, depression, and somatization symptoms. **Results:** The main predictors of poor mental health outcomes were sleeping less than six hours per night, inadequate protective equipment measures, female gender, personal and familiar infection, living alone, working as a nurse, and working in a COVID-19 ward. Healthcare workers in 2021 reported experiencing more serious accidents and stressful events than those of the first wave. Depressive symptoms and COVID-19-related adverse events were higher in the second pandemic outbreak than in the first. **Conclusions:** Preventive strategies against poor mental health outcomes should be particularly focused on female nurses who live alone, work in areas with high infection rates, and have experienced the COVID-19 infection personally or who are close to people that have experienced the infection.

## 1. Introduction

The coronavirus disease 2019 (COVID-19) pandemic placed extraordinary demands on healthcare systems, causing the rapid overwhelming of public health services and discontinuity of care in countries around the world (e.g., [1,2,3,4]). As a result, healthcare workers (HCWs) were exposed to an enormous amount of stress due to the high risk of infection and inadequate protection from contamination [5], the reassignment of professional duties, the extension of working shifts, the risk of social stigma, and the deprivation of/reduction in contact with their family members (e.g., [6]). 

One of the largest and most recent metanalyses [7] has documented the negative mental health consequences of the COVID-19 pandemic on HCWs, showing that post-traumatic stress disorder (PTSD) symptomatology (33%), sleep issues (37%), depression (23%), and anxiety (24%) symptoms were the most prevalent psychological difficulties. Another recent large-scale study from the UK [8] found that 21.5% of HCWs met the threshold for diagnosable mental disorders (assessed by clinical interviews), with an estimated population prevalence of PTSD of about 8% (95% Confidence Interval: 4.0–15.1%). Although the work environment is already linked to a fundamental aspect of HWCs’ mental health––namely, the perception of not wearing protective equipment measures, especially for those who work as frontline professionals and/or have direct contact with infected patients––the combination of various factors seems to have a major role [9,10]. Notably, other factors, such as being a nurse, having a part-time job [10,11,12], or being stigmatized because of augmented risk of exposure to COVID-19 at work [13], also seem crucial in increasing poor mental health outcomes. In addition, individual characteristics, such as being female, being younger, and having less professional experience, play a pivotal role in this context, potentially determining higher levels of distress in HCWs involved in the pandemic [10,14]. The fear of endangering the health of one’s family members and, thus, the need for prolonged separation from them have represented a significantly higher risk of psychological distress in HCWs [10]. Although some reports showed that HCWs’ mental health was more compromised in the post-acute outbreak than in the initial phase of previous pandemics (e.g., [15]), most of the existing studies have collected data from the first wave of the pandemic only (e.g., [11,16]). Monitoring the mental health outcomes of HCWs involved in the COVID-19 emergency and identifying associated predictors are key goals in regard to informing preventive strategies and interventions to mitigate current, future, and transgenerational adverse mental health outcomes.

The current cross-sectional study aims to identify potential predictors of poor mental health outcomes related to both work environment and individual characteristics in a large population of HCWs involved in the emergency in Italy. The evaluation of poor mental health outcomes was conceived in terms of somatization, depression, anxiety, and depersonalization/derealization symptoms, namely the most expected psychopathological reactions to stress and traumatic events, as also observed in previous studies [16]. Moreover, given the potential differences as a function of observed time frames [7,11], we compared data collected from two separate waves of the COVID-19 pandemic (April 2020 vs. June 2021), opening the possibility of investigating its long-term effects, particularly considering that the onset of mental disorders is a complex phenomenon which can occur later than expected. 

## 2. Materials and Methods

### 2.1. Study Design 

A snowball sampling technique was used to collect data during two separate waves of the COVID-19 pandemic by administering two online surveys to HCWs working in Italy. The data were gathered using a survey created on the Qualtrics platform (https://www.qualtrics.com/), which was distributed through a link shared via social networks. Participation was voluntary, and no incentives were provided. All participants gave their online informed consent under the Declaration of Helsinki (1964). The consent process was designed to ensure that participants fully understood the study’s objectives, procedures, and the assessment tools they would complete before starting the survey (i.e., it was presented as the first survey page). Examples of items from the assessment were provided to help participants gain a clear understanding of what their participation would involve. The cross-sectional study was approved by the Ethics Committee of the University of Padova (GDPR EU 2016, protocol number 4367).

The first sample completed the survey between 1 and 30 April 2020, which was during the first wave and, thus, during the peak of the COVID-19 pandemic in Italy, approximately five weeks after the beginning of the lockdown and just before the second stage of easing restrictions (e.g., to allow access to religious services, weddings, hairdressing services, and short-term hospitality without boarding). 

The second sample filled in the survey between 1 and 30 June 2021 during the second wave, in which the relative flattening of COVID-19 infection and the gradual release of government measures to limit infection in Italy occurred. 

### 2.2. Measures

Overall, the online survey included 90 items and took approximately 25 min to complete. 

The first section was designed to collect individual characteristics, such as sociodemographic characteristics (i.e., gender, age, region of residence), daily life aspects (i.e., numbers of sleeping hours, living alone/with others), work environment [professional category (i.e., physician, nurse, or other categories), workplace (i.e., public health unit, private health unit, general practitioner), working in areas with high infection rates (i.e., working in COVID-19 wards)], and availability of protective equipment measures.

#### 2.2.1. COVID-19-Related Adverse Events (CRAEs)

An ad hoc checklist composed of 5 items was developed to detect the frequency of CRAEs. The items refer to “infection among co-workers”, “death among co-workers”, “infection among family/friends”, “death among family/friends”, and “personal infection” rated on a dichotomous scale (i.e., Yes, No).

#### 2.2.2. Traumatic Life Events

The Life Events Checklist for DSM-5 (LEC-5) [17] was used to assess post-traumatic reactions to the COVID-19 pandemic. The LEC is originally based on a 17-item self-report measure evaluated on a 6-point nominal scale (i.e., “Happened to me”, “Witnessed it”, “Learned about it”, “Part of my job”, “Not sure”, “Doesn’t apply”) aimed at screening for potential lifetime traumatic events. It was developed by the National Center for PTSD and has demonstrated adequate psychometric properties in samples of undergraduates and combat veterans [18]. In the present study, we only included 5 items describing the most relevant events linked to the HCWs’ personal experiences (i.e., serious accidents at work, at home, or during recreational activities (item 4), the danger of death from illness or trauma (item 12), severe human suffering (item 13), sudden accidental death (item 15), and any other very stressful event or experience (item 17)), only going further for the sake of brevity and to ensure consistent respondent attention levels over time. A back-translation process has been employed in order to ensure the accuracy and cultural relevance of the translated items.

#### 2.2.3. Depression, Anxiety, and Somatization Symptoms

The depression (12 items), anxiety (9 items), and somatization (12 items) subscales of the Italian version of the Symptom Checklist-90-Revised (SCL-90-R) [19,20] were administered. Each item is rated on a 5-point Likert scale, from 0 (“Not at all”) to 4 (“Extremely”), where a value of >60 indicates the presence of psychopathological symptomatology. This scale was validated on a large community sample, yielding good internal consistency for all the subscales (Cronbach’s α between 0.70 and 0.96) [20]. 

#### 2.2.4. Depersonalization and Derealization

The Italian version of the 2-item version of the Cambridge Depersonalization Scale (CDS-2) [21,22,23] were used to assess the frequency of depersonalization (“Out of the blue, I feel strange, as if I were not real or as if I were cut off from the world”) and derealization (i.e., “My surroundings feel detached or unreal as if there was a veil between me and the outside world”) over the last two weeks (“Over the past two weeks, how often have you been bothered by...”). Each item was rated on a 4-point Likert scale from 0 (“Not at all”) to 3 (“Nearly every day”). The CDS-2 ≥ 3 cut-off determined clinically significant depersonalization/derealization with a sensitivity of 78.9% and a specificity of 85.7%. The CDS-2 has demonstrated high reliability (Cronbach’s α = 0.92) and was strongly correlated (r = 0.77) with a structured interview measuring the severity of depersonalization/derealization [21]. 

### 2.3. Statistical Analyses

#### 2.3.1. Descriptive Statistics

The variables considered in the present study were summarized in frequency tables and figures (frequencies were reported for each categorical variable, while median and interquartile ranges [IQRs] were reported for continuous variables). We computed nonparametric tests (i.e., Spearman’s rho correlations and Wilcoxon rank sum test) to compare the distribution of the considered variables on the ordinal Likert scale by sampling year (2020, 2021). Categorical variables were compared using the χ^2^ or Fisher’s exact test when the expected frequencies in any combination were less than 10. Statistical significance was assumed to be at the 5% level.

#### 2.3.2. Preliminary Analyses: Dimensionality Reduction—Exploratory Factor Analyses

As preliminary analyses, we performed EFAs to obtain an index summarizing poor mental health outcomes (i.e., depersonalization/derealization, anxiety, depression, and somatization symptoms, assessed by the SCL-90 and CDS-2 questionnaires). Given the non-normal distribution of this investigated domain, the EFA was carried out using a Weighted Least Square Mean and Variance adjusted (WLSMV) estimator. We adopted a variant of the likelihood ratio test [24] for investigating metric invariances between the two-time frames (i.e., 2020, 2021); since the metric invariance was not confirmed (*p* = 0.024), we estimated two different EFAs, one for each time period.

Then, we conducted a second dimensionality reduction based on EFAs to estimate an index that summarizes the occurrence of threatening events from a lifetime perspective, namely lifetime traumatic events (LTEs), by referring to items related to the presence of serious accidents at work, at home, or during recreational activities, the danger of death from illness or trauma, severe human suffering, sudden accidental death, and any other very stressful events (assessed by the LEC questionnaire). We followed the same statistical procedure as described above, but due to the presence of metric invariances by wave (*p* = 0.11), we performed a single EFA for the whole sample.

#### 2.3.3. Quantile Regression

Since the poor mental health outcomes index yielded an asymmetric distribution (Figure S1 in the Supplementary Materials), by setting up a quantile regression model based on the median (quantile = 0.5, i.e., the median value), we defined which factors played a role in determining this score. To find which predictors best described the data, a stepwise approach was used based on the Akaike Information Criterion (AIC) model selection strategy [25] (as it has already been implemented in previous research, e.g., [2,26]). Thus, in the model, we only included the predictive variables selected based on the AIC [27].

Specifically, the factors of gender (female vs. male), year of data collection (i.e., 2020 vs. 2021), age were divided into three groups, i.e. tertiles, based on the sample’s distribution of ages (i.e., ≤38, 38–50, >50 years), the medical health profession (physicians, nurses, other healthcare professionals), having worked in a COVID-19 ward, availability of protective equipment measures (i.e., weak or inadequate, adequate or sufficient), workplace (i.e., public health unit, private health unit, general practitioner), and average sleeping hours (i.e., ≤6 h, or >6 h). The region (Veneto, Lombardia, other regions) was included as an additional confounding factor.

Lastly, in the quantile regression model, we considered the effect of the LTE scores and CRAE scores. To investigate any differences in these latter factors among the two diverse time frames, we also tested while considering the AIC value, the presence of interactions between the above-mentioned regressors and the year of data collection (i.e., 2020, 2021). Model results were presented by coefficient estimates (β) and their relative 95% Coefficient Intervals (CIs). The quantile regression model was calculated using R 4.0 software by relying on the quantreg package [28].

## 3. Results

### 3.1. Descriptive Differences in the Two-Year Data Collection

Out of N = 557 respondents, n = 411 were enrolled in 2020 and n = 146 were enrolled in 2021. The overall sample showed a marked predominance of females (78%) and a median age of 44 years (IQR = 33–52), ranging from 21 to 77 years. Respondents frequently reported living with other relatives or cohabitants (75%) and were mostly nurses (53%), followed by physicians (24%) and other healthcare professionals (23%). Enrolment involved mainly two Italian regions (Veneto 48% and Lombardia 38%), with a high frequency of practitioners being employed by a public health facility. 

Slightly more than half reported having worked in a COVID-19 ward, and a high prevalence of respondents reported sufficient (40%) or adequate (32%) availability of personal protective equipment measures against COVID-19 infection. In addition, most HCWs reported sleeping between 6 and 8 h per night (42%) or less than 6 h (25%). 

Considering the years in which the survey took place, some marginally significant differences emerged between the two subsamples (see Table 1). In particular, in 2021, compared to 2020, the respondents came mainly from the Lombardia region (*p* < 0.001) and there was an increase in the number of people working in a COVID-19 ward (*p* < 0.001) and the availability of protective equipment measures (*p* = 0.002).

### 3.2. Preliminary Analyses: Dimensionality Reduction—Exploratory Factor Analyses Results 

The EFAs employed to summarize the poor mental health outcomes explained a satisfactory variance quote of 65% for 2020 and 64% for 2021. The two indices showed close estimated values, with a slight increase in the loadings related to anxiety and somatization symptoms in 2021 compared to 2020. The regression factor scores were calculated for the entire dataset using the estimated loadings. 

For the second EFA performed to summarize the occurrence of LTEs, as before, we extracted the first factor, which explains a discrete quote of variance (30%), as the score related to the intensity of potentially traumatic events and assessed it using the LEC questionnaire. 

Figure S1 and Table S1 (Supplementary Materials) show for each EFA the number and the name of the items included, their internal consistency (Cronbach’s α), the estimated loadings, and the proportion of deviance explained.

### 3.3. Differences in Symptoms in the Two-Year Data Collection

**Figure 1:**
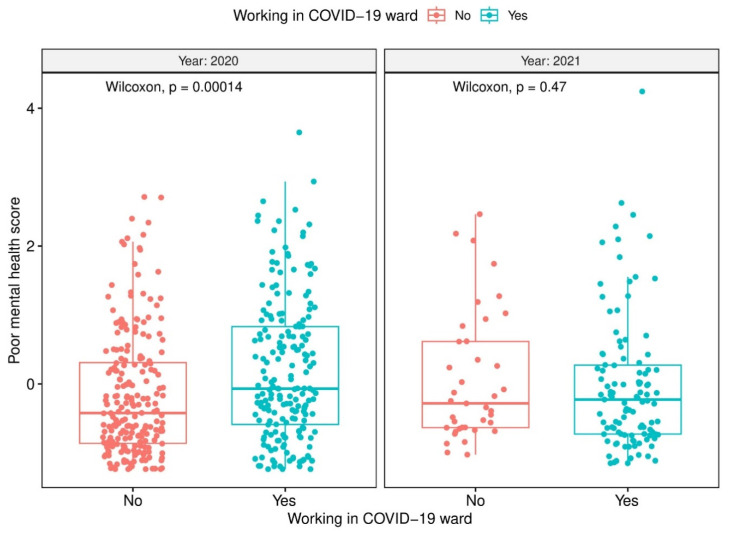
Factorial scores of the poor mental health outcomes index by work in a COVID-19 ward and year of the data collection (2020 vs. 2021).

Table 2 shows the distribution and differences in scales related to poor mental health outcomes, LTEs, and CRAEs by year of data collection. Specifically, participants in 2021 reported higher depressive symptoms than those interviewed in 2020 (*p* < 0.001). In terms of LTEs, respondents in the 2021 group experienced more serious accidents and stressful events in general (both *p* < 0.001) compared to respondents in 2020; however, they reported having witnessed human suffering to a lesser extent (*p* < 0.001). All variables related to CRAEs increased in 2021 compared to 2020, particularly those related to infection among co-workers in a COVID-19 ward and among family and friends. Figure 1 displays the distribution of the poor mental health outcomes index based on having worked at a COVID-19 ward as a function of the year of data collection, reporting an increase in the score only among those enrolled in 2020.

Figures S2 and S3 (Supplementary Materials) reported the distribution and Spearman’s rho correlations between the scales of depersonalization/derealization, anxiety, depression, and somatization symptoms, and between LTEs, respectively.

### 3.4. Quantile Regression Results

Table 3 reports the estimated coefficients of the quantile regression. Overall, poor mental health outcomes decrease on the median by 0.62 (95%IC: 1.17–0.08) among those who were surveyed in 2020 (vs. 2021), by 0.30 points (95%IC: 0.50–0.10) among females (vs. males), by 0.22 points (95%IC: 0.39–0.05) among those living alone (vs. with co-habitant), and by 0.18 (95%IC: 0.34–0.03) among nurses (vs. physicians). 

Moreover, all individual and COVID-19 pandemic-related variables reported a significant effect in increasing poor mental health outcomes in the overall sample. In particular, a strong effect on this score was observed in those who slept less than six hours per night compared to those who slept more than six hours (β: 0.57; 95%IC: 0.39–0.75), when protective equipment availability was weak or inadequate (β: 0.34 points, 95%IC: 0.03–0.49), and in cases of personal infection (β: 0.30, 95%IC: 0.15–0.45). In addition, working in a COVID-19 ward shows a small effect on poor mental health outcomes (β: 0.15 points, 95%IC: 0.03–0.27). 

Five significant interaction terms between covariates and the year of collection were included: being male and living with others proved to be positive predictors against poor mental health outcomes in 2021 with respect to 2020, while working as a nurse and having an infection among family/friends resulted in negative predictors of poor mental health outcomes in 2021 with respect to 2020.

## 4. Discussion

This cross-sectional study investigated potential predictors of poor mental health outcomes among HCWs in Italy while comparing data collected from two separate waves of the COVID-19 pandemic (April 2020 vs. June 2021).

Overall, results showed that the main negative predictors of poor mental health outcomes are associated with individual characteristics, factors related to the work environment, and personal experiences associated with the COVID-19 pandemic. 

In regard to individual characteristics, being female, living alone, and working as a nurse were found to be negative predictors of poor mental health outcomes. The relationship between being female and working as a nurse is consistent with recent meta-analytic evidence [29], which demonstrated that female HCWs are predominantly nurses. This can, in part, explain our results, since nurses are those who have experienced the disease first-hand and are directly involved in patient care, therefore potentially experiencing more persistent psychological pressure and exposure to the threat of COVID-19 infection [30,31]. In addition, being a nurse might also be associated with a lower socioeconomic status, which has been previously shown to be a negative predictor of psychological distress in the female Italian population [32]. 

Furthermore, the impact of living alone on the HCWs’ poor mental health could suggest that daily interpersonal bonding with family members could decrease the feelings of isolation and discrimination often reported by HCWs. Indeed, previous evidence showed that social support decreased emotional exhaustion or burnout [33,34], representing a positive predictor of poor mental health despite the fear of infecting their close ones [35]. In line with this, our results showed that having experienced the contagion effecting family members or friends, or having been infected themselves, were additional negative predictors of poor mental health outcomes. In addition, poor sleep hours (less than six per night), working in a COVID-19 ward, and having inadequate protective equipment measures at work represent negative predictors of poor mental health outcomes. Our findings are consistent with previous research showing that prolonged work in a COVID-19 ward was significantly associated with negative mental health outcomes [35]. Sleep disorders, particularly insomnia, were commonly reported in frontline HCWs in hospitals during the COVID-19 pandemic and were often associated with depressive and anxiety symptoms [35,36]. 

More specifically, in 2020, compared to 2021, nurses reported more poor mental health outcomes than physicians and other healthcare professionals. However, in 2021 compared to 2020, this trend changed, and nurses reported fewer poor mental health outcomes than physicians and other healthcare professionals. These results may suggest that, at first, physicians generally tend to underestimate their psychological distress symptoms (for a review, see [37]) compared to nurses; at the same time, nurses may have adapted to the situation, no longer perceiving a state of psychological suffering. Moreover, HCWs who participated in the first survey in 2020, compared to those who took part in 2021, exhibited higher levels of depressive symptoms. This finding could be interpreted by referring to several negative factors which were more prevalent in 2021, including being employed as a physician [38], having worked in a COVID-19 ward [31,38], and having a more significant number of infected family members and friends [39,40,41]. However, it should also be considered that the difference in response rates between the two waves might potentially affect the comparability of these factors across the two periods.

The significant increase in depressive symptoms reported by the HCWs in 2021 could also be considered a delayed response to the cumulative stress they had encountered since the beginning of the COVID-19 emergency, confirming a trend in line with what happens months after being exposed to potentially traumatic life events [42]. This finding is also consistent with those obtained in studies conducted on HCWs in 2003 after the SARS outbreak in Taiwan, where a more significant amount of depressive symptomatology was reported rather than hyperarousal responses (e.g., anxiety, distress) in the later stage of the crisis, when the infection was deemed more manageable and under control [15]. In addition, our results showed that a history of frequent traumatic events played a role in the HCWs’ poor mental health, having a greater impact in 2021 than in 2020. This finding could be explained by the sudden and rapid escalation of the epidemic in 2020, with the corresponding depletion of healthcare resources and staff to cope with the emergency [43,44], which may have led to greater psychological distress. 

Although 2021 showed a better scenario than 2020, as suggested by the decreased death rates both within the workplace and outside, it should be considered that there are also positive predictors of poor mental health outcomes. These latter elements include being less involved in emergencies and perceiving more safety with every possible protective medical measure, which is a predictable sign of the healthcare system being better prepared to deal with the COVID-19 pandemic [45].

### Strengths and Limitations

Our cross-sectional/naturalistic design does not allow us to draw actual causal conclusions about the impact of the COVID-19 pandemic on the psychological adaptation of HCWs. A partial remedy was the analysis based on a regression model adjusted for a series of potential confounders. However, the comparison of separate data collected during different waves of the COVID-19 pandemic is informative; prospective cohort or interrupted series designs allow for the clarification of the relationships between the considered variables. Second, a selection bias is related to the presence of a convenience sample of HCWs involved in this study. Participants who chose to complete the surveys might be those particularly in need of sharing their experience during the pandemic and therefore more prone to actively reflect on it. Furthermore, most responses were concentrated in two regions, Veneto and Lombardia, which were the most severely affected by the pandemic in Italy [46]. While this focus provided critical insights into the experiences of HCWs in the hardest-hit areas, it may limit the generalizability of our findings to other regions. Notwithstanding this, considering the entirely voluntary and anonymous setting in which our data were collected, this should be considered a minor concern.

## 5. Conclusions

The present study shed light on predictors that contribute to the onset and/or exacerbation of poor mental health outcomes in response to the two challenging COVID-19 waves in an Italian sample of HCWs, depicting diversified scenarios based on the period of the examined emergency. According to our findings, detection, monitoring, and preventive/treatment strategies should be particularly focused on HCWs who are female, work as nurses, live alone, work in areas with high infection rates (COVID-19 wards), and who experienced a personal infection or who were close with someone who experienced an infection. Depressive symptoms may be a more distal post-traumatic reaction, as they occurred mostly a year later, thus making them a potential target for current mental health interventions. These results suggest that emotion regulation interventions, better work conditions, and assurance might help support and increase mental health outcomes while mitigating the adverse impacts of the COVID-19 pandemic within the HCW population. 

## 6. Relevance for Research and Clinical Practice

The empirical findings of this study highlighted the importance of understanding the mental health state of HCWs, which should be protected by, for instance, ensuring adequate sleep periods and the availability of adequate infection protection measures. Consistently, the global clinical research community is making significant efforts in this direction, also driven by substantial research investments deployed to establish effective treatments for physical and mental health outcomes in HCWs, further leveraging the potential of digital solutions, such as eHealth and telemedicine [43,44,45]. Further research is needed to fully understand how these technologies can be tailored to meet the specific needs of HCWs, particularly in high-stress environments like pandemics, and to ultimately ensure their optimal implementation. As reported by [45], there is a need for countries to activate preventive measures to support the mental health of HCWs by, on the one hand, offering psychological support, such as telephone helplines and remote counseling. On the other hand, a welfare system in which working time adjustments, financial compensation, childcare facilities, and work support measures (e.g., free transport, housing, and continuing education credits) are guaranteed should be introduced. Future works should explore the long-term effectiveness of these preventive measures and their impact on the overall mental health, work–home balance, and job satisfaction of HWCs.

## Figures and Tables

**Table 1:** Individual characteristics of the overall sample by year of data collection.

	Overall N = 557 ^1^ (2020–2021)	Year of Data Collection		*p*-Value ^2^
		n = 411 ^1^ (2020)	n = 146 ^1^ (2021)	
Gender				0.68
Female	432 (78%)	317 (77%)	115 (79%)	
Male	125 (22%)	94 (23%)	31 (21%)	
Age	44 (33, 52)	45 (33, 53)	41 (32, 50)	0.003
Living alone				0.67
Yes	94 (17%)	71 (17%)	23 (16%)	
No	463 (83%)	340 (83%)	123 (84%)	
Medical Health Profession				0.067
Physicians	135 (24%)	108 (26%)	27 (18%)	
Nurses	292 (53%)	204 (50%)	88 (60%)	
Other healthcare professionals	130 (23%)	99 (24%)	31 (21%)	
Region of residence				<0.001
Veneto	267 (48%)	252 (61%)	15 (10%)	
Lombardia	209 (38%)	94 (23%)	115 (79%)	
Other regions	81 (15%)	65 (16%)	16 (11%)	
Working in a COVID-19 ward	302 (54%)	195 (47%)	107 (73%)	<0.001
Availability of personal protective equipment measures				0.002
Adequate	179 (32%)	118 (29%)	61 (42%)	
Sufficient	221 (40%)	161 (39%)	60 (41%)	
Weak	106 (19%)	91 (22%)	15 (10%)	
Inadequate	51 (9.2%)	41 (10.0%)	10 (6.8%)	
Place of work				0.053
Public Health Unit	429 (77%)	307 (75%)	122 (84%)	
Private Health Unit	107 (19%)	85 (21%)	22 (15%)	
General practitioner	21 (3.8%)	19 (4.6%)	2 (1.4%)	
Average sleeping hours				0.79
<6	139 (25%)	99 (24%)	40 (27%)	
(6–8)	233 (42%)	171 (42%)	62 (42%)	
(8–10)	98 (18%)	75 (18%)	23 (16%)	
≥10	87 (16%)	66 (16%)	21 (14%)	

Note. ^1^ Median (IQR) or Frequency (%); ^2^ Wilcoxon rank sum test; Pearson’s χ^2^ test.

**Table 2:** Descriptive statistics and Wilcoxon’s rank sum test related to poor mental health outcomes, levels of lifetime traumatic events (LTEs) and presence of COVID-19-related adverse events (CRAE), overall and by year of data collection.

Poor Mental Health Outcomes	Overall N = 557 ^1^ (2020–2021)	Year of Data Collection		*p*-Value ^2^
		n = 411 (2020)	n = 146 (2021)	
Depersonalization/derealization	0.00 (0.00, 2.00)	0.00 (0.00, 2.00)	0.00 (0.00, 2.00)	0.28
Anxiety	0.70 (0.30, 1.20)	0.70 (0.30, 1.20)	0.70 (0.30, 1.17)	0.60
Depression	0.77 (0.31, 1.46)	0.69 (0.31, 1.38)	0.92 (0.48, 1.69)	0.004
Somatization symptoms	0.58 (0.25, 1.08)	0.58 (0.25, 1.12)	0.58 (0.25, 1.08)	0.42
LTEs				
Serious accidents (at work, home, or during recreational activities)	2.00 (1.00, 3.00)	2.00 (0.00, 3.00)	3.00 (3.00, 5.00)	<0.001
The danger of death from illness or trauma	3.00 (2.00, 3.00)	3.00 (2.00, 3.00)	2.00 (2.00, 4.00)	0.21
Severe human suffering	3.00 (2.00, 3.00)	3.00 (2.00, 3.00)	2.00 (2.00, 2.00)	<0.001
The sudden accidental death	3.00 (2.00, 4.00)	3.00 (2.00, 4.00)	3.00 (1.00, 5.00)	0.083
Any other very stressful events	3.00 (1.00, 4.00)	2.00 (0.00, 4.00)	3.00 (1.00, 5.00)	<0.001
CRAE				
Infection co-workers	496 (89%)	350 (85%)	146 (100%)	<0.001
Death co-workers	219 (39%)	133 (32%)	86 (59%)	<0.001
Infection family/friends	467 (84%)	327 (80%)	140 (96%)	<0.001
Death family/friends	135 (24%)	76 (18%)	59 (40%)	<0.001
Personal Infection	65 (12%)	29 (7.1%)	36 (25%)	<0.001

Note. ^1^ Median (IQR) or Frequency (%); ^2^ Wilcoxon rank sum test; Pearson’s χ^2^ test.

**Table 3:** Adjusted * estimated coefficients and a relative 95% Confidence Interval (95%CI) of a quantile regression regarding the year of data collection, individual characteristics, CRAEs on poor mental health outcomes, and their interactions, concerning the reference category **.

Predictors	β	95%CI	*p*-Values
(Intercept)	−0.30	−0.58–−0.03	0.032
Year of data collection (2021)	−0.62	−1.17–−0.08	0.025
Individual characteristics			
Gender (Male)	−0.30	−0.50–−0.10	0.003
Age-class (38–50]	0.04	−0.11–0.19	0.594
Age-class [51–77]	−0.13	−0.27–0.02	0.100
Living alone (No)	−0.22	−0.39–−0.05	0.012
Job employment (Other)	−0.17	−0.35–0.00	0.054
Job employment (Physician)	−0.18	−0.34–−0.03	0.023
Working in a COVID-19 ward (Yes)	0.15	0.03–0.27	0.017
Availability of personal protective equipment measures (weak or inadequate)	0.34	0.19–0.49	<0.001
Sleeping hours (less than 6)	0.57	0.39–0.75	<0.001
CRAEs			
Death co-workers (Yes)	0.13	−0.01–0.26	0.066
Infection among family/friends (Yes)	0.11	−0.05–0.26	0.193
Personal infection (Yes)	0.30	0.15–0.45	<0.001
Year (2021) * Gender (Male)	−0.36	−0.63–−0.08	0.011
Year (2021) * Age-class (38–50]	−0.32	−0.65–0.01	0.058
Year (2021) * Age-class [51–77]	−0.24	−0.48–0.01	0.056
Year (2021) * Job employment (Other)	0.36	0.07–0.65	0.015
Year (2021) * Job employment (Physician)	0.41	0.10–0.71	0.009
Year (2021) * Infection family/friends (Yes)	0.59	0.06–1.11	0.028
Observations	557		
R^2^	0.193		

Note. * Adjusted also for the region of residence (Veneto, Lombardia, Other Regions), ** Reference categories: gender (Female), year of data collection (2020), age class (≤38), living alone (Yes), work in a COVID-19 ward (No), availability of personal protective equipment measures (adequate or sufficient), sleeping hours (more than 6), personal infection (No), infection family/friends (No), death co-workers (No), job employment (Nurse).

## Data Availability

The raw data supporting the conclusions of this article will be made available by the authors on request.

## Supplementary Materials

The following supporting information can be downloaded at https://www.mdpi.com/article/10.3390/healthcare12191921/s1. Figure S1. Marginal distribution of the poor mental health outcomes index. Table S1: Explorative Factor Analyses performed for the estimation of poor mental health outcomes and a score of lifetime traumatic events (LTEs). Figure S2: Distribution and Spearman’s rho correlation between scales of depersonalization/derealization, anxiety, depression, and somatization symptoms; Figure S3: Pairwise distribution and Spearman’s rho correlations between groups of lifetime traumatic events (LTEs).
