# Supplementary material for: Predictors of Poor Mental Health Outcomes in Healthcare Workers during COVID-19: A Two Waves Study
Source: Healthcare (Basel). 2024 Sep 25;12(19):1921. doi: 10.3390/healthcare12191921 (PMC11476261; doi:10.3390/healthcare12191921)
Supplement: Supplementary file 1 [file healthcare-12-01921-s001.zip › healthcare-3156892-supplementary.pdf]

Supplementary Materials

Figure S1. Marginal distribution of the poor mental health outcomes index.

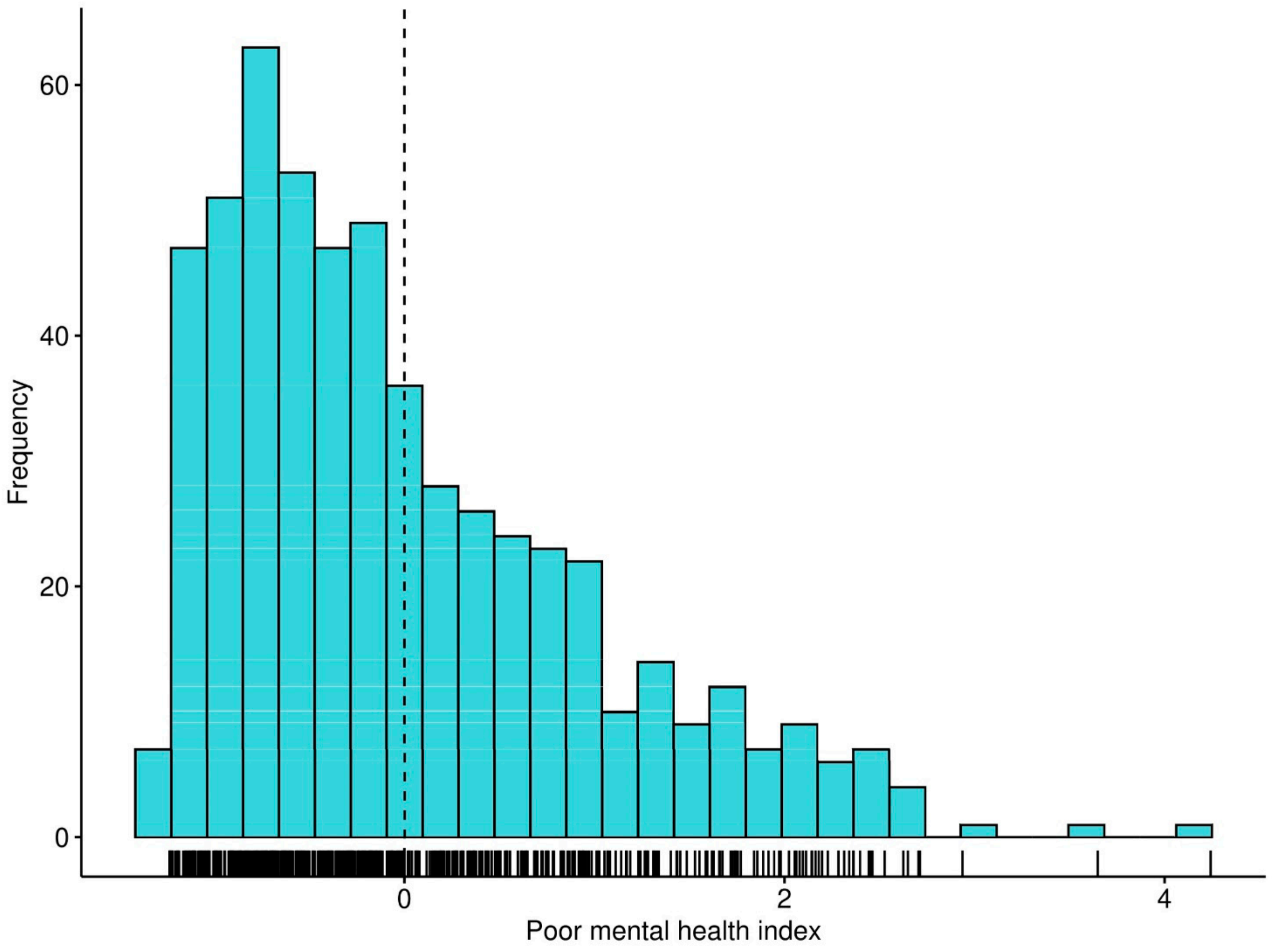

Table S1. Explorative Factor Analyses performed for the estimation of poor mental health outcomes and a score of lifetime traumatic events (LTEs).

	**Poor mental health outcomes index**		**Lifetime Traumatic Events (LTEs)**
N. of items	4	4	5
N	411	146	557
Cronbach's α	0.81	0.80	0.64
Item 1	Depersonalization/ derealization	Depersonalization/ derealization	Serious accidents
Item 2	Anxiety	Anxiety	Danger of death from illness or trauma
Item 3	Depression	Depression	Severe human suffering
Item 4	Somatization symptoms	Somatization symptoms	Sudden accidental death
Item 5	-	-	Any other very stressful events
Load. Item 1	0.63	0.59	0.47
Load. Item 2	0.93	0.95	0.78
Load. item 3	0.91	0.88	0.54
Load. item 4	0.70	0.75	0.45
Load. item 5	-	-	0.41
SS loadings	2.59	2.57	1.50
% var. explained	65	64	30

Figure S2. Distribution and Spearman's rho correlation between scales of depersonalization/derealization, anxiety, depression, and somatization symptoms.

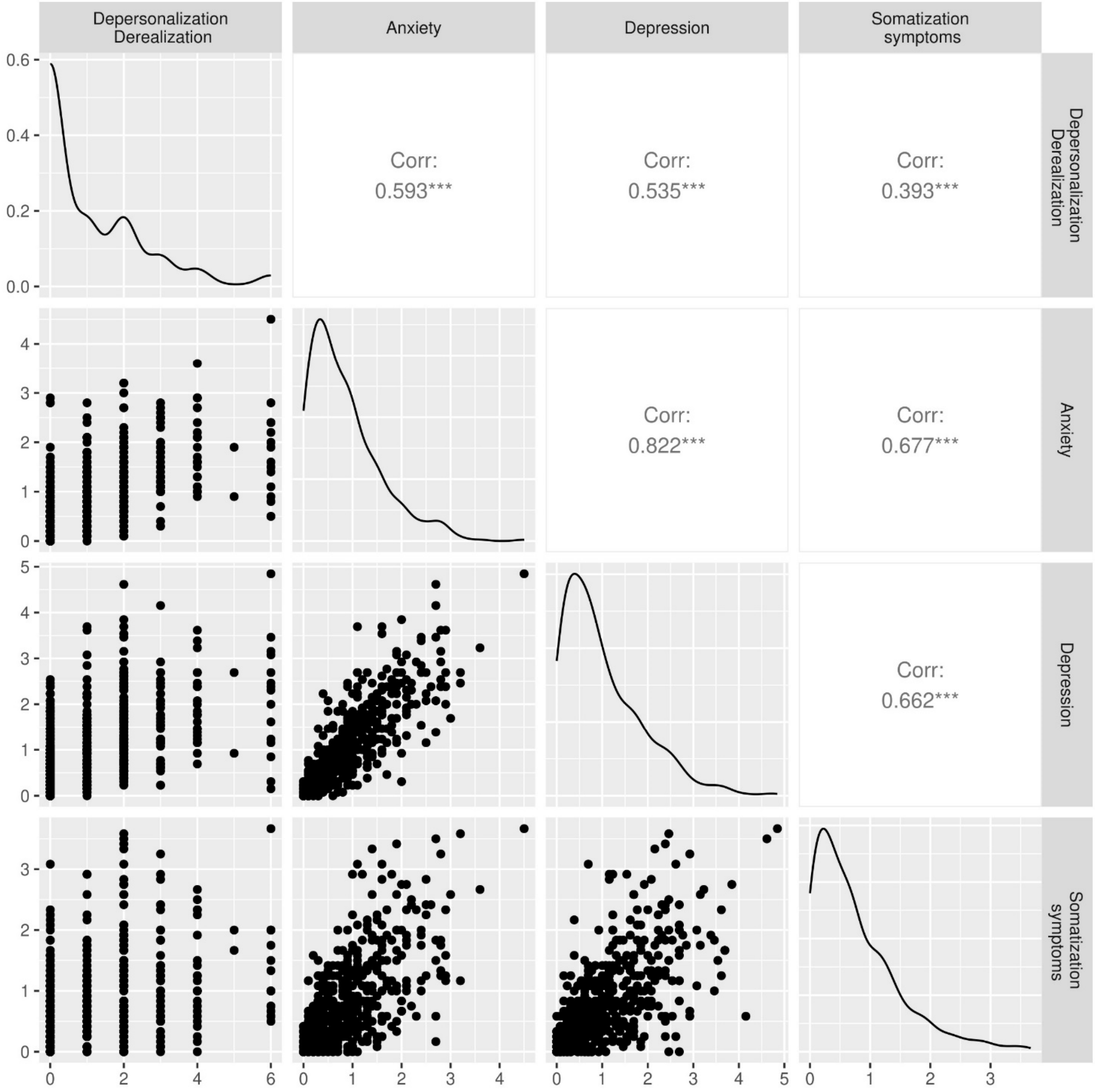

Note. N = 557; ***p < 0.001, **p < 0.01,*p < 0.05

Figure S3. Pairwise distribution and Spearman's rho correlations between groups of lifetime traumatic events (LTEs).

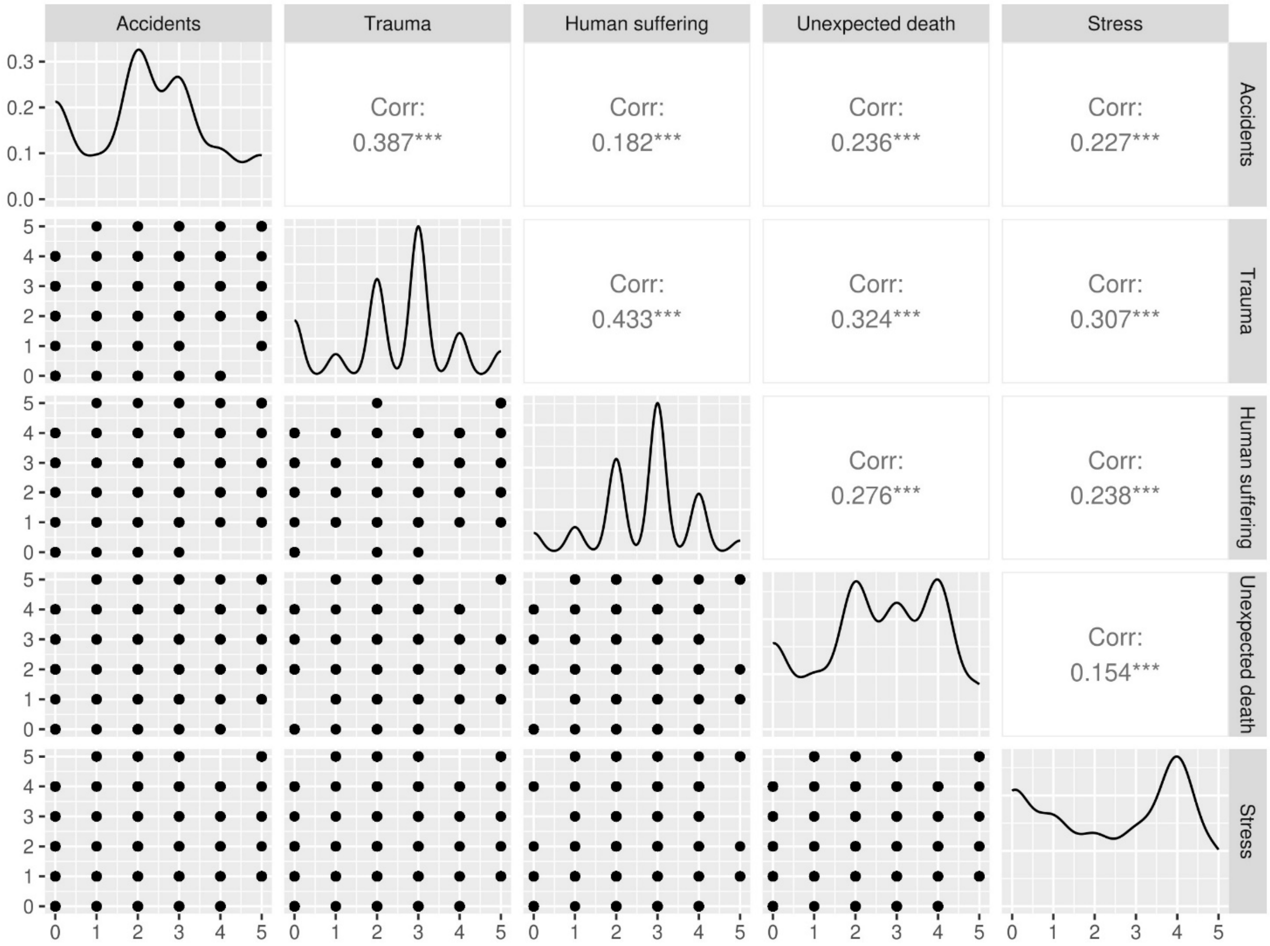

Note. N = 557; ***p < 0.001, **p < 0.01,*p < 0.05
